# The T Cell Receptor Repertoire in Neuropsychiatric Systemic Lupus Erythematosus

**DOI:** 10.3389/fimmu.2020.01476

**Published:** 2020-07-17

**Authors:** Erica Moore, Michelle W. Huang, Shweta Jain, Samantha A. Chalmers, Fernando Macian, Chaim Putterman

**Affiliations:** ^1^Department of Microbiology and Immunology, Albert Einstein College of Medicine, Bronx, NY, United States; ^2^Early Discovery and Fundamental Research, Hansoh Bio, Rockville, MD, United States; ^3^Department of Pathology, Albert Einstein College of Medicine, New York, NY, United States; ^4^Division of Rheumatology, Albert Einstein College of Medicine, Bronx, NY, United States; ^5^Bar-Ilan University Azrieli Faculty of Medicine, Ramat Gan, Israel; ^6^Galilee Medical Center, Nahariya, Israel

**Keywords:** systemic lupus erythematosus, MRL/lpr, T cell receptor, neuropsychiatric lupus, choroid plexus, salivary gland

## Abstract

**Objective:** In systemic lupus erythematosus (SLE), widespread T cell infiltration into target organs contributes to inflammation and organ damage. Autoreactive T cells become aberrantly activated in this disease due to dysfunctional T cell receptor signaling that lowers the activation threshold. Characterizing the T cell repertoire can provide further insight into the specific homing and proliferation of these T cells into lupus target organs. In the spontaneous lupus model, MRL/lpr, the TCR repertoire has not been fully elucidated, especially for T cells infiltrating the brain. Our aim was to investigate and compare the TCR repertoire between MRL/lpr mice and its congenic controls, MRL/MpJ, and within MRL/lpr tissues.

**Methods:** Spleen, salivary gland, and brain choroid plexus were isolated from female MRL/lpr mice and MRL/MpJ mice. The TCRβ CDR3 region was analyzed by multiplex PCRs and sequencing.

**Results:** Significant differences were seen not only between the MRL/lpr and MRL/MpJ spleens, but also between MRL/lpr tissues. The TCR repertoire in MRL/lpr choroid plexus tissues had significantly increased clonality and sequence homology compared to MRL/lpr spleen and salivary gland. The consensus sequence, CASSQDWGGYEQYFF, was identified in the MRL/lpr choroid plexus repertoire.

**Conclusions:** The TCR repertoire in lupus prone mice is not uniform between target organs, and suggests that T cells are specifically recruited into the choroid plexus of MRL/lpr mice. Further studies are needed to determine the antigen specificities for these infiltrating T cells in target organs of lupus mice, and their possible contribution to the pathogenesis of neuropsychiatric disease and other lupus manifestations.

## Introduction

Systemic lupus erythematosus (SLE) is an autoimmune disease characterized by the formation of autoantibodies and immune complexes, and the presence of autoreactive T cells. In SLE, these autoreactive T cells are integral in disease pathogenesis by propagating inflammatory cytokine secretion, facilitating autoantibody formation via B cell help, and differentiating into autoreactive memory T cells (1–5). These inflammatory effects are further exaggerated by the aberrant infiltration of these T cells into organs, thus causing subsequent damage and injury. In SLE, many tissues are commonly affected including the kidneys and the skin. The central nervous system is another prominent organ system involved, whose manifestations such as cognitive dysfunction and depression affect 20–40% of SLE patients (6).

T cells recognize a complex of human leukocyte antigen (HLA) molecules and antigenic peptides, which mediates the interaction between T cells and antigen-presenting cells. In particular, the complementarity-determining region 3 (CDR3) loops are positioned to interact with the presented antigenic peptide and is considered to be the most diverse segment. This recognition leads to the activation and proliferation of antigen-specific T cells (7). Alterations in the T cell receptor (TCR) repertoire have been found in multiple autoimmune diseases, including SLE, rheumatoid arthritis, and Type 1 diabetes mellitus, and are implicated in the breakdown of peripheral immune tolerance (8–14). Analyzing the repertoire of clonally expanded T cells can potentially reveal specific homing of these T cells based on local antigen-driven activation.

The pathogenesis of neuropsychiatric SLE (NPSLE) is complex and incompletely understood. Mouse models have proven particularly valuable in elucidating the pathogenesis of target organ inflammation and damage in lupus, including NPSLE. The MRL/lpr mouse strain, named for the extensive lymphoproliferation, is a spontaneous mouse model that parallels many of the manifestations (i.e., anti-dsDNA autoantibodies, immune complex deposition) seen in human SLE patients, as well as diffuse neuropsychiatric manifestations such as cognitive deficits and depressive-like behavior (15, 16). Both the MRL/MpJ strain (the parent strain) and MRL/lpr mice have the same genetic background that predisposes to autoimmune disorders, but the MRL/lpr strain has an accelerating homozygous Fas mutation, further promoting the loss of self-tolerance. In the MRL/lpr strain, T cells can be found infiltrating into the choroid plexus, which forms the blood-cerebrospinal fluid barrier (17). The role of T cells in neuropsychiatric lupus has not been fully elucidated. Moreover, based on the heterogeneous manifestations seen, the T cell response in SLE is likely not driven by a singular peptide epitope but rather widespread targets that have not yet been identified and may likely not be uniform across patients.

In this study, we characterized choroid plexus-infiltrating T cells in neuropsychiatric lupus by performing TCRβ CDR3 sequencing and comparing the TCR repertoire to the salivary gland and spleen of MRL/lpr mice and the spleen from its control strain, MRL/MpJ. We not only demonstrate significant differences between the MRL/lpr and MRL/MpJ strains, but also between MRL/lpr tissues.

## Materials and Methods

### Animals

Female MRL/MpJ-Fas^lpr/lpr^ (MRL/lpr) and MRL/MpJ-Fas^+/+^ (MRL/MpJ) mice were either purchased from the Jackson Laboratory at 5–8 weeks of age or bred at Albert Einstein College of Medicine. Mice were housed at 21–23°C on a 12-h light/12-h dark cycle. All animal protocols were approved by the Albert Einstein College of Medicine Institutional Animal Care and Use Committee.

### Tissue Isolation

MRL/lpr (*n* = 9) and control MRL/MpJ (*n* = 5) mice were sacrificed between 17 and 21 weeks of age. Mice were perfused with PBS (Corning, Corning, NY) and tissues including spleen, salivary gland (submandibular), and choroid plexus were isolated. Tissues were snap frozen until DNA isolation could be performed in batches.

### Immunofluorescent Staining

MRL/lpr mice were sacrificed at 16 weeks of age and brains and salivary glands were fixed in 2.5% paraformaldehyde for 24 h at 4°C. The paraformaldehyde was subsequently diluted to 1% until paraffin embedding, sectioning, and staining could be performed. Sections of the paraffin-embedded salivary glands and coronally-cut brains were blocked with 20% normal horse serum. These were then incubated with primary rabbit anti-mouse CD4 and rat anti-mouse CD8 antibodies (1:100, Sino Biological, Wayne, PA; eBioscience, San Diego, CA), followed by a secondary AF488-conjugated donkey anti-rabbit antibody and Cy5-conjugated donkey anti-rat antibody (1:200, Jackson ImmunoResearch, West Grove, PA). Fluorescence-stained slides were imaged under an Invitrogen microscope (Invitrogen EVOS FL Auto 2 Cell Imaging System) and were analyzed using ImageJ (National Institutes of Health, Bethesda, MD).

### Flow Cytometry

Single cell suspensions were generated from MRL/lpr tissues including spleen, lymph node, choroid plexus, submandibular salivary glands, lungs, and kidney. Briefly, tissue was excised, cut into small pieces, and placed in a microcentrifuge tube with a digest enzyme working solution of 20 ug/mL Liberase TL (Roche, Basel, Switzerland) and 2 units/mL DNase (Roche) in RPMI at 37°C, shaking for 30 min. The reaction was quenched, followed by centrifugation and resuspension. Cells were incubated with anti-mouse CD16/32 antibody (BD) on ice for 30 min for Fc receptor blocking. Surface staining for CD45, CD3, CD4, and CD8 was performed, and the samples were run on a BD LSRII. Data was collected with FACS Diva software and analyzed with FlowJo_V10 software (TreeStar, Ashland, OR, USA).

### DNA Isolation

DNA was isolated from the samples in two batches with the DNEasy Blood and Tissue Kit (Qiagen, Germantown, MD). The DNA concentration and quality were evaluated by Nanodrop (Thermo Fisher Scientific, Basingstoke, United Kingdom).

### TCRβ Sequencing

TCRβ sequencing was performed using the Adaptive Biotechnologies immunoSeq assay, as previously described (18). In brief, the samples underwent multiplexed PCR gene amplification and then amplicons were sequenced with Illumina HiSeq. The input material for lymphoid samples was 800 ng, while ≥ 1,000 ng was used to increase the number of TCR sequence captures in non-lymphoid samples.

### TCR Sequence Homology

Sequences were entered into the IEDB Epitope Cluster Analysis (iedb.org) to identify consensus sequences. Parameters included at least 70% sequence identity threshold and a peptide length cutoff of 9 amino acids. The “cluster-break for clear representative sequence” algorithm was used for the actual clustering.

### Antigen Specificity

Sequences were entered into the McPAS-TCR curated database (friedmanlab.weizmann.ac.il/McPas-TCR/) to identify matching sequences.

### Data and Statistical Analysis

TCR repertoire diversity metrics were analyzed with either Adaptive Biotechnologies immunoSeq Analyzer V.3.0 and the R package, LymphoSeq. Statistics were performed with Graphpad Prism 7 software (La Jolla, CA). Multiple comparisons were assessed by one-way ANOVA, with Bonferroni's correction when appropriate. *P* < 0.05 were considered statistically significant.

## Results

### The TCR Repertoire Is Clonally Expanded in MRL/lpr Spleens Compared to MRL/MpJ Spleens

The MRL/lpr strain develops extensive lymphoproliferation due to an accelerating Fas mutation, further promoting the loss of self-tolerance, compared to the parental strain MRL/MpJ which only develops autoimmune features much later in life (19). Therefore, we initially compared the TCR repertoires in MRL/lpr vs. MRL/MpJ mice with diversity metrics.

**Figure 1:**
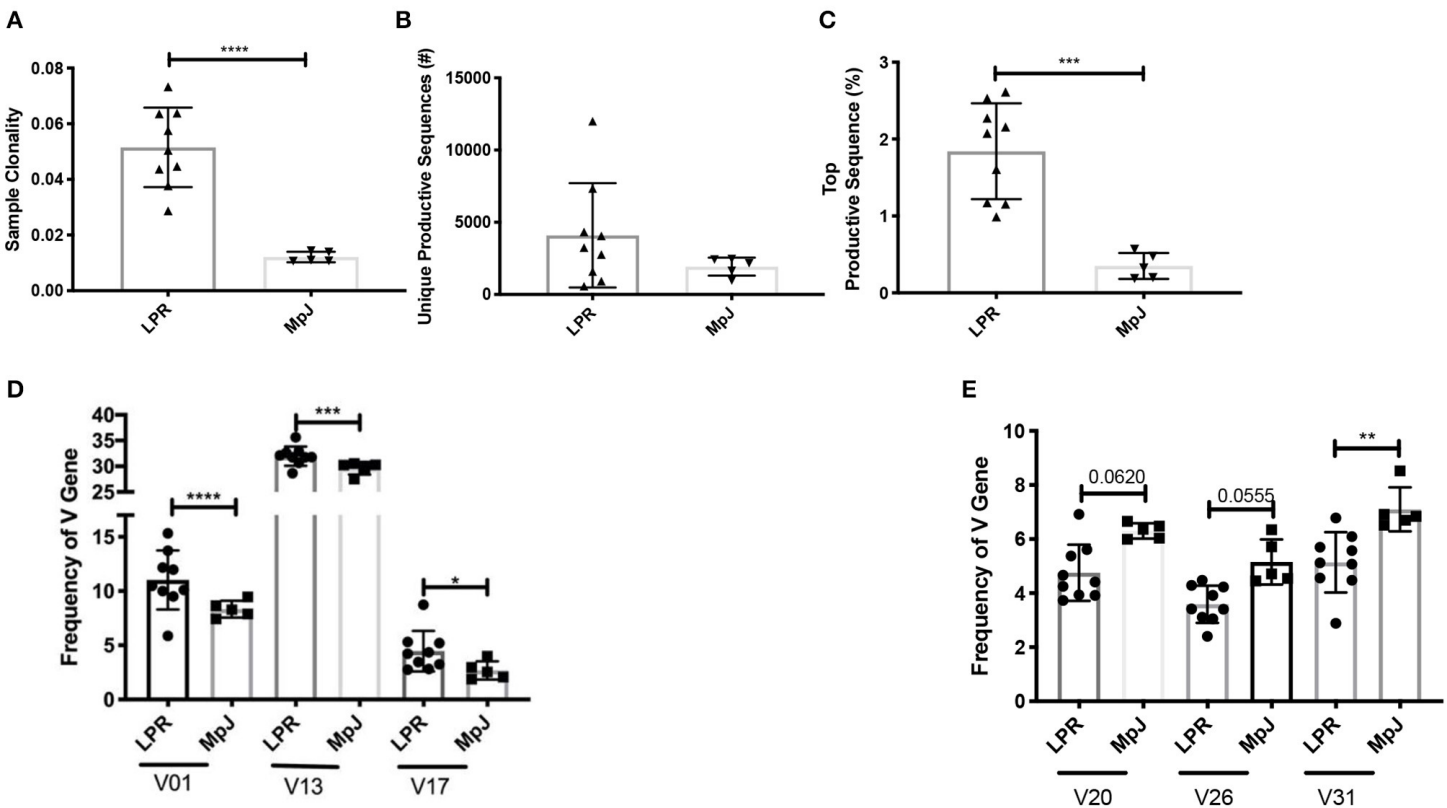
Comparison of the TCR repertoire between MRL/lpr and MRL/MpJ spleens. The TCR repertoires of MRL/lpr (*n* = 9) and MRL/MpJ (*n* = 5) spleens were analyzed. **(A)** Sample clonality. **(B)** Number of unique productive TCR clones. **(C)** Frequency of the top productive TCR clones. **(D)** Selective increased TCRB V gene usage in MRL/lpr spleen repertoires compared to MRL/MpJ. **(E)** Specific decreased TCRB V gene usage in MRL/lpr spleen repertoires compared to MRL/MpJ. Each dot indicates one mouse and bar graphs represent mean ± SD. *P*-values were determined with an unpaired *T*-test, *p < 0.05, **p < 0.01, ***p < 0.001, ****p < 0.0001.

Clonality reflects the expansion of specific TCR clonotypes and reveals the T cell immune response to recognition of locally presented antigens. MRL/lpr splenic tissue demonstrated increased clonality compared to the MRL/MpJ strain (Figure 1A). There was a trend for an increased number of unique productive sequences as well as a significant increase in the frequency of the top productive rearrangements in MRL/lpr spleens than in MRL/MpJ (Figures 1B,C, respectively). This increased clonality indicates that even with the lymphoproliferation characteristic of this strain, the TCR repertoire present in the MRL/lpr spleen is oligoclonal. When comparing the repertoires' TCRβ V gene usage, the most prevalent gene segment in both MRL/MpJ and MRL/lpr strains was TCRβ V13. However, the MRL/lpr spleen samples had increased diversity in TCRβ V gene usage, with significantly enriched usage of the Vβ genes V01, V13, and V17 (Figure 1D). In contrast, TCRβ V20, 26, and 31 genes were enriched in the MRL/MpJ repertoires (Figure 1E).

### T Cells With Distinct TCR Repertoires Infiltrate Into Different MRL/lpr Organs

**Figure 2:**
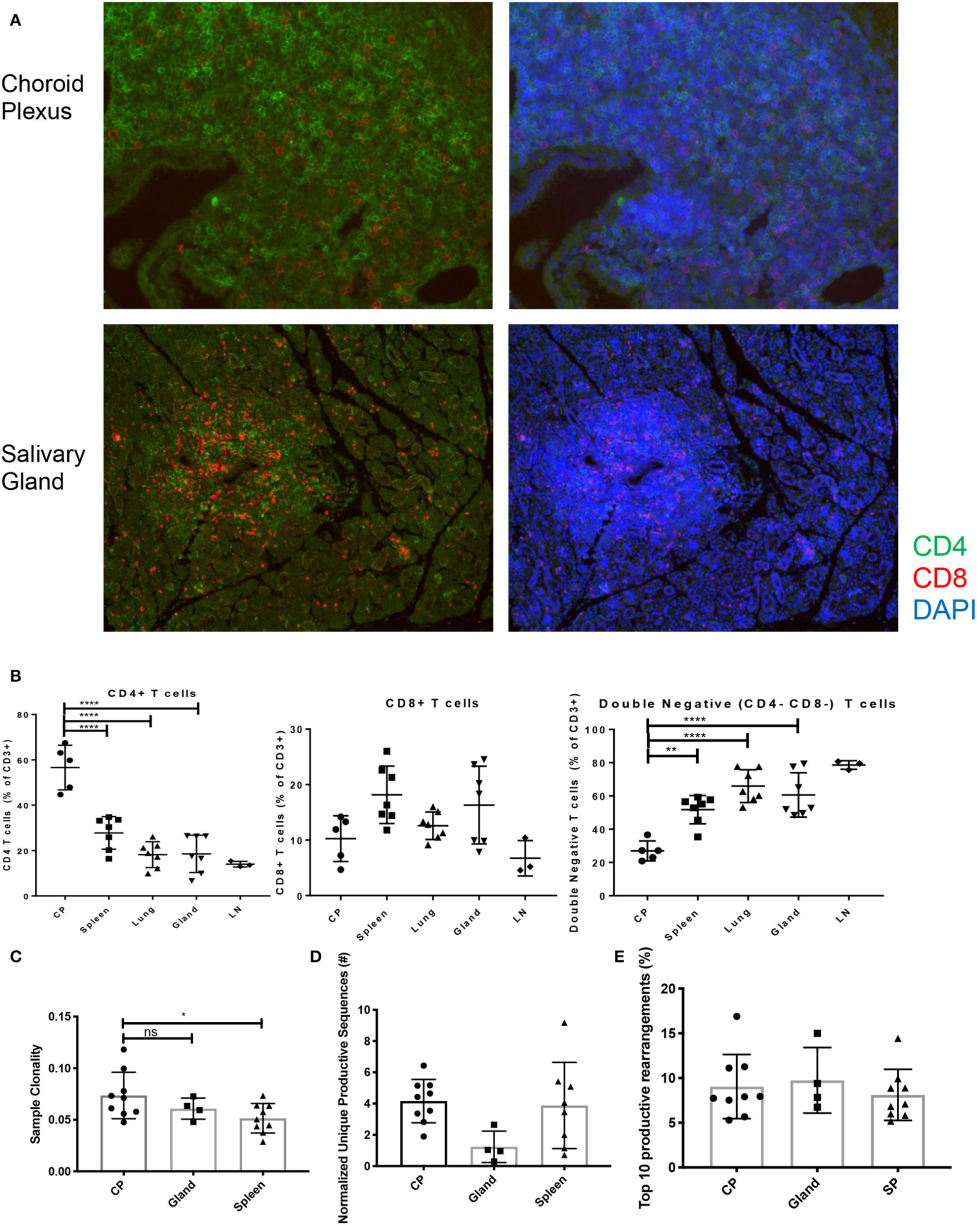
T cell infiltration and subsets in MRL/lpr tissues. **(A)** Immunofluorescent staining for CD4 (green) and CD8 (red) T cell infiltration in choroid plexus (top) and salivary gland (bottom), magnification = 20X. **(B)** Percentage of CD4+ T cells (left), CD8+ T cells (center), and double negative T cells (right) in MRL/lpr tissues by flow cytometry; CP, choroid plexus; Gland, salivary gland; LN, lymph node. Values represent percentages of parent CD3+ T cells. **(C)** Sample clonality of MRL/lpr spleen (*n* = 9), salivary gland (*n* = 4), and choroid plexus tissues (*n* = 9). **(D)** Normalized number of unique productive clones in MRL/lpr tissue repertoires calculated by dividing the total number of unique productive clones to the amount of DNA analyzed. **(E)** Sum of the top 10 productive rearrangement frequencies. *P-*values were determined with one-way ANOVAs, *p < 0.05, **p < 0.01, ****p < 0.0001.

Mimicking SLE disease in humans, T cells can be found infiltrating into several target organs including kidney, skin, and brain in MRL/lpr mice. Additionally, the MRL/lpr model is also used to study the pathogenesis of Sjogren's disease, an autoimmune disease that predominantly affects the salivary glands (19). The function of brain-infiltrating T cells in the pathogenesis of NPSLE, however, has not been clarified. Both CD4 and CD8 T cells can be seen infiltrating into the brain, specifically the choroid plexus, and the salivary glands (Figures 2A,B). Notably, the composition of the T cell infiltrates in the choroid plexus is quite different from either the spleen or the salivary gland, supporting a specific recruitment process of T cells into the choroid plexus (Figure 2B). To gain insight into the clonality of these cells, we performed TCRβ sequence analysis on spleen, salivary gland, and choroid plexus tissues from MRL/lpr mice.

The choroid plexus had an increased degree of oligoclonality when compared to the MRL/lpr splenic tissue but not to MRL/lpr salivary gland tissue (Figure 2C). To account for the different amounts of DNA analyzed between lymphoid and non-lymphoid tissue, the normalized number of unique productive clones showed that there was no difference between the MRL/lpr choroid plexus and spleen repertoires, but the salivary gland trended to have a decreased amount (Figure 2D). When comparing the frequency of the top 10 productive rearrangements, there was no significant difference between the tissues (Figure 2E). However, when additional productive rearrangements (i.e., top 50) were included, the spleen had several clones that were enriched at a higher frequency but otherwise displayed a more diverse repertoire comparable to the other two tissues (data not shown).

### Choroid Plexus-Infiltrating T Cells Preferentially Use TCRβ V 2, 4, 12, and 24

**Figure 3:**
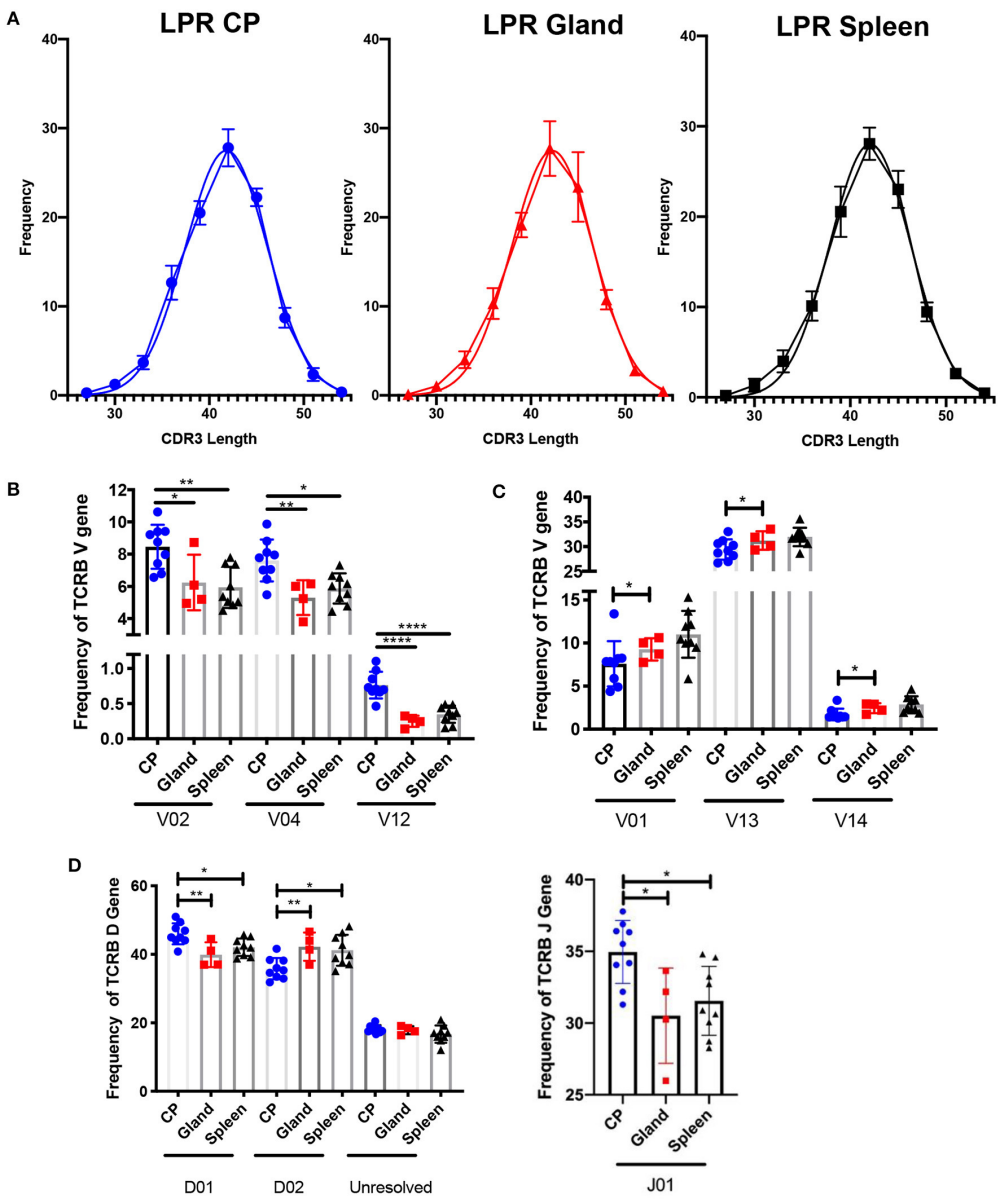
The choroid plexus exhibits distinct TCR profiles compared to other MRL/lpr tissues. **(A)** Frequency of CDR3 lengths compared to Gaussian distributions in MRL/lpr choroid plexus (left), salivary gland (middle), and spleen (right) TCR repertoires. **(B)** Selective increased TCRB V gene usage in MRL/lpr choroid plexus tissue. **(C)** Specific decreased TCRB V gene usage in MRL/lpr choroid plexus tissue. **(D)** Frequency of TCRB D (top) and J gene (bottom) usage in MRL/lpr tissues. *P-*values determined by one-way ANOVAs, *p < 0.05, **p < 0.01, ****p < 0.0001.

Another metric for assessing diversity in the T cell repertoire is the length of the CDR3 loop. In the MRL/lpr strain, the length of the CDR3 loop in the spleen, salivary gland, and choroid plexus appeared similar to an expected Gaussian curve (Figure 3A), and not skewed to either shorter or longer CDR3 lengths.

V gene usage, however, was notably variable between these three tissues. The choroid plexus T cell repertoire showed significant enrichment in Vβ 2, 4, and 12 genes compared to the splenic and salivary gland tissues (Figure 3B), and in Vβ26 relative to salivary gland alone (not shown). In contrast, Vβ genes 1, 13, and 14 were decreased in the choroid plexus compared to the other tissues (Figure 3C). The choroid plexus also diverged from the other MRL/lpr tissues in its enriched use of the Dβ1 gene and Jβ1 gene, compared to preferential use of Dβ2 and Jβ2 in the salivary gland and spleen (Figure 3D). The skewed gene usage seen implies that specific T cell clones are infiltrating and expanding in the choroid plexus.

### Shared Clones in MRL/lpr Choroid Plexus Tissues Demonstrate Homology

**Figure 4:**
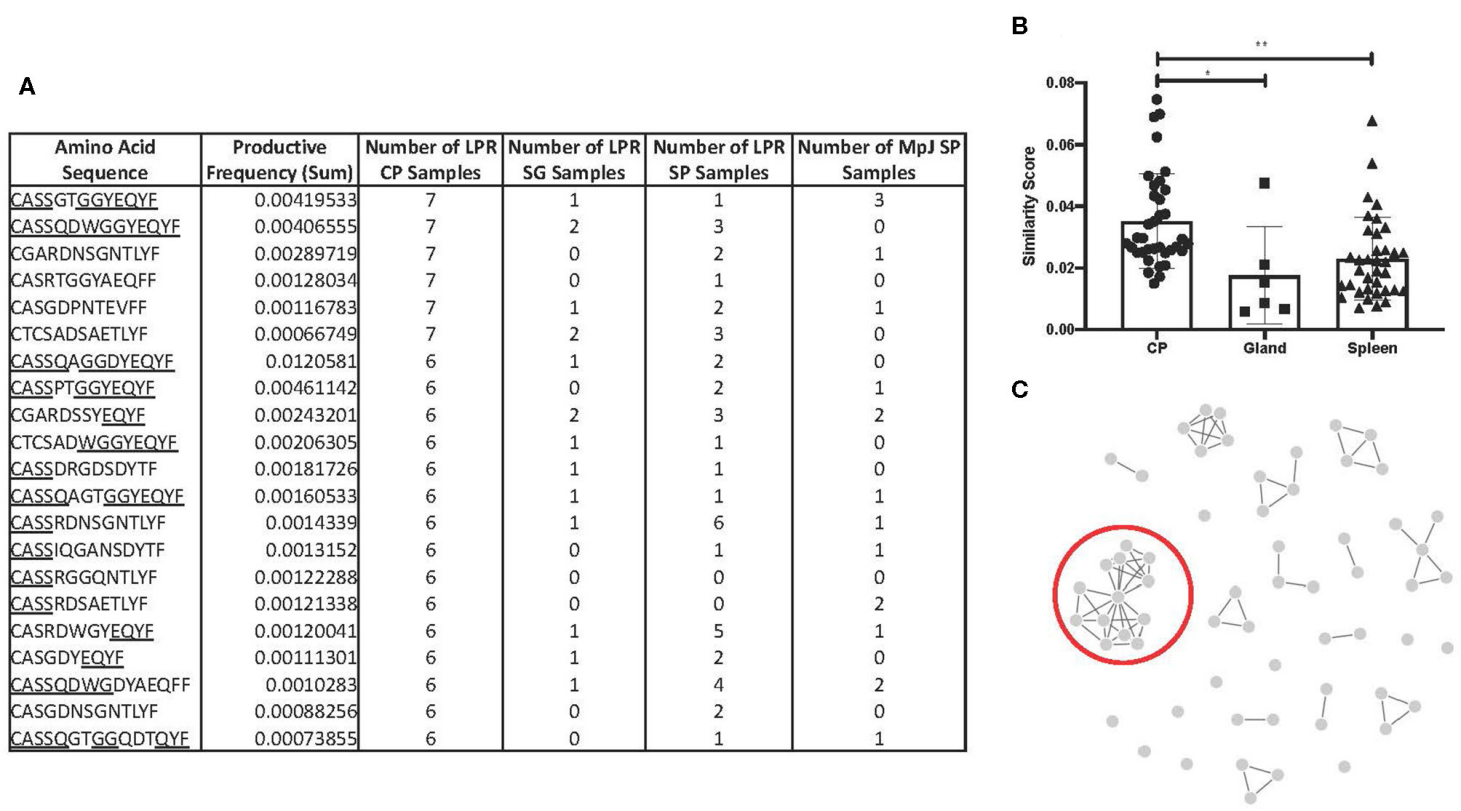
TCR sequence homology in the MRL/lpr choroid plexus. **(A)** Overlapping TCR sequences in ≥ 6 MRL/lpr choroid plexus tissues compared to MRL/lpr splenic and salivary gland tissues and MRL/MpJ splenic tissues. Underlined residues indicate matching amino acid sequences to identified consensus sequence, CASSQDWGGYEQYFF. **(B)** Similarity scores, measuring the amount of overlap between two TCR repertoires, generated within each MRL/lpr tissue. **(C)** Visual representation of epitope cluster analysis (IEDB.org) of identified TCR sequences with 80% overlapping. Red circle indicates sequences similar to the consensus sequence identified. LPR CP = MRL/lpr CP tissues, LPR SG = MRL/lpr salivary gland tissues, LPR SP = MRL/lpr spleen tissues, MpJ SP = MRL/MpJ spleen tissues. *P-*values calculated with one-way ANOVA, *p < 0.05, **p < 0.01.

To counter the extensive array of pathogens that T cells might encounter, TCR sequences in humans and mice are diverse, and do not commonly overlap in sequence homology between one individual to the next. Interestingly, we identified 21 amino acid sequences that were found in six or more MRL/lpr choroid plexus tissues (Figure 4A). These particular sequences, with the exception of CASSRDNSGNTLYF and CASRDWGYEQYF in the MRL/lpr splenic repertoire and CASSGTGGYEQYF in the MRL/MpJ splenic repertoire, were not prominently reflected in either salivary glands or spleen tissue from MRL/lpr and MRL/MpJ strains.

We generated a similarity score, which uses Jaccard similarities to calculate a pairwise CDR3 similarity matrix based on the frequencies of CDR3 motifs. Among the MRL/lpr choroid plexus repertoires, there was significantly increased homology and higher similarity scores between the samples (Figure 4B). In fact, when we expanded the list of amino acid sequences to those present in at least half the MRL/lpr choroid plexus samples (5 out of 9), we were able to identify clusters of sequences that had at least 70% sequence homology. The most prominent cluster had a consensus sequence of CASSQDWGGYEQYFF (Figure 4C).

Having sequenced the TCRβ CDR3 region in the MRL/lpr lupus strain, we compared our repertoire to those previously published with known antigen specificities. Using the McPAS-TCR curated database and the sequences from Figure 4A, we only found 3 of the 21 sequences in the database: CASSQDWGGYEQYF, CASSRDSAETLYF, and CASGDYEQYF, which matched sequences identified in murine diabetes type 1 NOD/ShiLtJ and C57/Bl6J influenza models.

## Discussion

The role of T cells in neuropsychiatric lupus is not sufficiently understood, despite both the presence of brain-infiltrating T cells in both NPSLE models and human NPSLE patients (17, 18). T cell repertoires offer insight into the adaptive immune response in disease, as each TCR is unique and the selection of few over many can be used as a tool to inform disease mechanisms. The clonal expansion of T cells indicates a local presentation of antigenic peptides, which in the case of autoimmunity, implies the selection and proliferation of autoreactive T cells. Advances in sequencing technology, such as TCRβ sequencing, have enabled both greater ease and detail when characterizing TCR repertoires. For autoimmune diseases like SLE, the TCR repertoire can potentially be used for identifying the widely unknown peptide targets, but also for diagnostic and monitoring purposes.

In the present study, we have used TCRβ sequencing to characterize the TCR repertoires in choroid plexus-infiltrating T cells in a neuropsychiatric lupus mouse model. The MRL/lpr is a well-established spontaneous lupus model that mimics human systemic disease manifestations, including anti-dsDNA autoantibody production, immune complex deposition, and aberrant T cell function, and diffuse neuropsychiatric manifestations including cognitive deficits and depressive-like behavior. Since both human SLE and the MRL/lpr show neuropsychiatric manifestations, and choroid plexus lymphoid infiltrates are common in the MRL/lpr strain and have been described in human lupus, (15, 16, 20–24) we used this mouse model to readily assess the brain and other organs for TCRβ sequencing.

Although MRL/lpr mice display prominent neuropsychiatric manifestations, one possibility to consider is that T cells are randomly infiltrating the brain, as a part of the lymphoproliferation in this strain and the highly vascularized nature of the choroid plexus where the T cell accumulation is most prominent. In selecting appropriate organs for comparison, we included splenic tissue from both MRL/lpr and MRL/MpJ mice to represent the systemic T cell repertoire in each strain. In addition, MRL/MpJ mice serve as a background control for the lupus strain. In MRL/lpr mice we chose to sequence the TCRβ repertoire of the salivary gland, since similar to the choroid plexus this tissue contains tertiary or ectopic lymphoid structures (23). Moreover, the MRL/lpr mouse model is frequently used to study mechanisms of disease in Sjögren's syndrome, as this strain displays prominent T cell infiltration and eventually destruction of the salivary glands (25). The differing percentages of CD4, CD8, and double negative T cells across a number of MRL/lpr tissues, in and of itself, indicates that CD4 and CD8 infiltration into the choroid plexus is specific and is likely a directed process. Support for this hypothesis can be found in previous studies of the distribution of T cell subsets in the kidneys of MRL/lpr mice. Similar to what we described here for the choroid plexus, infiltration of nephritogenic T cells in this strain is associated with a significant decrease in double negative T cells and increase in the percent of CD4+ T cells, (26) as well as the specific recruitment of clonal and pathogenic T cells into different compartments in the kidneys (27).

We found that T cells from MRL/lpr tissues were more oligoclonal than MRL/MpJ mice. We also showed that the T cells in the choroid plexus in MRL/lpr mice have skewed V gene usage, including Vβ 2, 4, and 12, compared to autologous splenic and salivary gland tissue. Furthermore, the strongest degree of sequence homology in all of the MRL/lpr tissues were the TCRs in the choroid plexus as they had significantly higher similarity scores. We identified the consensus sequence, CASSQDWGGYEQYFF, out of clones overlapping the majority of choroid plexus samples. While the sequences in the choroid plexus did not match exactly, it reasonable to assume that different T cell clones arose with similar specificities.

Reduction in TCR diversity is correlated with disease activity in a number of autoimmune diseases, including SLE. The abnormal cloning of TCR sequences is implicated in the loss of peripheral tolerance seen in autoimmune diseases. In previous human SLE studies a biased V gene usage has been reported, including the expansion of Vβ12 (12). Additionally, it was recently shown that the TCR repertoire can be used diagnostically to differentiate SLE from rheumatoid arthritis patients or healthy controls. The authors suggested that the TCR profile could be used as a biomarker in a lieu of serological indicators (28). A similar finding in the biased usage of Vβ13 was previously identified in MRL/lpr spleens, (29) which we observed as well.

A recent study by Morawski and Bolland detailed the TCR repertoire of non-pathogenic CD8+ T cells in the brains of TLR7-transgenic (TLR7-tg) mice, another model for murine lupus (30). In this repertoire, the authors concluded that there was increased oligoclonality and homology and skewed VDJ gene usage, including a graphical increase in Vβ02 and Dβ01 and a decrease in Vβ13, which parallels our findings in the choroid plexus repertoire of MRL/lpr mice. Interestingly, the dominant clone in one of the TLR7-tg mice studied, CASSLGRRIYEQYF, has similar sequence motifs to the consensus sequence identified in the MRL/lpr choroid plexus, perhaps implicating a similar antigen specificity. However, differing from our MRL/lpr TCR repertoire, the abundance of CDR3 length in the repertoire present in the TLR7-tg strain, particularly the brain, diverted from Gaussian distribution. The differences in the TCR repertoire between these two cohorts could be the result of a number of factors including different pathogenic mechanisms in these two strains, different composition of T cell infiltration, or the restriction to CD8+ clones compared to both CD4 and CD8 included in this MRL/lpr cohort. Characterizing the MRL/lpr repertoire in the choroid plexus offers novelty, both as a comparison to the TLR7-tg model, but also as a widely established neuropsychiatric lupus model that exhibits both behavioral deficits and neuropathology.

While choroid plexus-infiltrating T cells may be reacting to brain specific antigens, this has yet to be proven. We did not find any matches in the TCR repertoire database that were brain antigens. Because these databases are manually curated, it is possible that the reactivity of these top clones has not been studied before. With certain parameters, there are emerging methods, such as TCRmodel, that could use CDR3 sequences from the alpha and beta chains to construct a predicted TCR structure and antigen peptide reactivity, (31) which we plan to employ in future studies. An additional potential weakness of our approach is that the repertoire contains a mixture of CD4 and CD8 T cells. Future studies aimed at directly comparing the choroid plexus CD4 and CD8 TCR repertoire would be interesting, and help in identifying the brain antigens recognized by choroid plexus T cells and predicting the relevant TCR structures. Since MHC I and MHC II present peptides of different lengths to CD8+ and CD4+ T cells, respectively, the dominant clones in each T cell subset may very well differ. On the other hand, if the repertoires are similar, this would support an interpretation that the choroid plexus infiltrating CD4 and CD8 T cells recognize the same autoantigenic locus. With additional advances in sequencing such as single cell RNA sequencing with immune profiling, evaluating both the TCR diversity and T cell phenotypes simultaneously could delineate CD4 and CD8 TCR sequences separately, and provide data for TCR modeling and MHC prediction. While CD4 and CD8 T cells can be separated cytometrically and the TCR repertoire then compared between these subsets, the very small size of the choroid plexus which was the primary tissue of interest in our present study precluded this approach. Similarly, while it would be compelling to compare the choroid plexus TCR repertoire across different time points in MRL/lpr mice, this was precluded due to the amount of choroid plexus material.

In conclusion, organ-infiltrating T cells in MRL/lpr mice have a distinct TCR repertoire. Of particular interest are those infiltrating the choroid plexus, as their role in the pathogenesis of NPSLE has not yet been fully elucidated; therefore, detailing the clonally expanded TCR repertoire might provide crucial insight into the pathogenesis of this key lupus manifestation. The unique profile of TCRβ sequences in the choroid plexus strongly suggests that these clonotypes are specifically recruited to this tissue and that likely selective pressures in the choroid plexus, like antigen presenting cells, are shaping the diversity of TCRs present. Specifically, the consensus sequence identified in the choroid plexus TCR repertoire is CASSQDWGGYEQYFF. The enrichment of the TCRβ repertoires in the choroid plexus may assist in dissecting the mechanisms contributing to neuropsychiatric lupus, and potentially reveal a novel therapeutic target.

## Data Availability Statement

The raw CDR3 sequencing data supporting the conclusions in this article will be made available by the authors, without reservation, for any legitimate research purpose.

## Ethics Statement

All animal studies were performed under protocols approved by the Institutional Animal Care and Use Committee of the Albert Einstein College of Medicine.

## Author Contributions

EM, MH, SJ, SC, FM, and CP conceived and designed the experiments. EM and MH performed the tissue excision and DNA isolation. EM analyzed the data. All authors wrote and/or edited the article and approved the final submitted version.

## Conflict of Interest

The authors declare that the research was conducted in the absence of any commercial or financial relationships that could be construed as a potential conflict of interest.
